# Prevalence of Stroke in Asian Patients with Sickle Cell Anemia: A Systematic Review and Meta-Analysis

**DOI:** 10.1155/2021/9961610

**Published:** 2021-06-03

**Authors:** Sandip Kuikel, Robin Rauniyar, Sanjeev Kharel, Anil Bist, Subarna Giri, Sahil Thapaliya, Sunanda Paudel

**Affiliations:** ^1^Maharajgunj Medical Campus, Tribhuvan University Institute of Medicine, Maharajgunj, Kathmandu 44600, Nepal; ^2^Department of Neurology, Tribhuvan University Teaching Hospital, Kathmandu 44600, Nepal

## Abstract

Sickle cell anemia (SCA) is an inherited autosomal recessive disease. It is caused due to point mutation that substitutes glutamate with valine at the sixth amino acid position of the beta chain of hemoglobin molecules leading to the sickling of the red blood cells and decreased structural deformability. Silent cerebral infarcts are the most common neurological complication of SCA, while overt stroke comprises substantial burden in patients with SCA. This meta-analysis aimed to find the pooled prevalence of overt stroke in SCA patients and discuss the importance of screening them. PubMed, Embase, and Google Scholar were the electronic databases used to search the studies. A total of 765 articles were retrieved upon detailed searching in the abovementioned databases. After a series of removing duplicate articles, title and abstract screening, and full-text review, 20 articles were found eligible and included in the study. The total number of participants from all the included studies was 3,956, and pooled prevalence of stroke in patients with sickle cell anemia in Asia was found to be 5% (95% CI: 4%, 6%) with a range from 1 to 41%. Stroke occurrence in sickle cell anemia patients is an emergency complication that needs immediate intervention and management. Because of the high prevalence of stroke in patients with sickle cell anemia, clinicians should focus on its prevention and treatment strategies.

## 1. Introduction

Sickle cell disease is a group of inherited red blood cell disorders that affects the oxygen-carrying protein, hemoglobin; of which, the most common type is known as sickle cell anemia (SCA). It results in alteration in the shape and function of hemoglobin. This alteration subsequently results in a lifetime of hemolytic anemia that is often complicated by vaso-occlusion of various sites [1]. The genetic basis of the disease was first discovered in 1958 and was found to be the substitution of glutamate with valine at the sixth amino acid position of the beta chain of hemoglobin molecules. This point mutation results in a change in the hemoglobin molecule leading to the sickling of the red blood cells in their deoxygenated state [2]. This sickling of RBCs makes them break down prematurely that causes hemolytic anemia. The reduced deformability of RBCs causes them trapped inside small vessels that eventually causes vaso-occlusive crises, including serious complications like stroke, acute chest syndromes, and renal failure [1].

Mutation in the globin chain-forming HbS is the most common pathological hemoglobin mutation worldwide [3]. It is estimated that about 7% of the world's population is a carrier, and about 300,000–500,000 babies are born each year with severe forms of the disease [3, 4]. The greatest burden of this disease is seen in Africa and Asia [5].

Neurological complications of SCA include seizures and coma, sinovenous thrombosis, reversible posterior leukoencephalopathy, or acute demyelination [6]. Though silent cerebral infarcts are the most common neurological complications of SCA, overt stroke comprises substantial burden in patients with SCA [7–9]. The pathophysiology of stroke in such patients includes not only the occlusion of blood vessels by trapping of sickled blood vessels but also the hypercoagulable state. This hypercoagulable state is caused due to an increased level of thrombin and a decreased level of proteins C and S. Furthermore, it can cause flow-related injury to the endothelial cells causing increased adhesiveness of sickle cell to them and may lead to stroke [10].

In this article, we systematically review articles highlighting the prevalence of stroke in patients with sickle cell anemia in Asia and estimate the pooled prevalence of overt stroke in them. This review tried to summarize the prevalence of stroke in SCA patients in Asia as the greatest burden of SCA is seen in Africa and Asia, and no study has reported it from Asia. We have emphasized on knowing the epidemiology and not on the interventions. The number thus found by this study helps public health personnel focus on developing measures to prevent stroke and decrease the risk of stroke substantially in patients with SCA.

## 2. Materials and Methods

### 2.1. Search Strategy and Selection Criteria

This study was done in accordance with the Preferred Reporting Items for Systematic Reviews and Meta-Analyses (PRISMA) statement. First of all, a search strategy was developed, and three of the major databases that include PubMed, Embase, and Google Scholar were searched separately. All studies from 2000 to 2020 were included in the study.

In our search, 175 studies were obtained from PubMed, 438 studies from Embase, and 152 studies from Google Scholar. Our systematic review was not previously registered with any of the international systematic review registers.

The search strategy used for Embase is given in Appendix 1. A total of 628 studies were identified after removing duplicates. The staged screening was performed; first, title and abstract screening were done.

Inclusion criteria for our study were as follows:

- Study done in the Asian continent
- Sickle cell anemia patients
- Patients developing stroke in sickle cell anemia
- Both ischemic and hemorrhagic stroke
- Prevalence data available
- Observational studies
- Diagnosis of stroke and sickle cell anemia by standard methods

Exclusion criteria for our study included the following:

- Case reports, meta-analysis, editorials, and systematic review
- Full text not available
- Prevalence data not available in the study
- Not a favourable outcome such as patients with silent infarcts

Title and abstract screening were done by two authors (Kuikel S and Giri S) using Covidence. The third author (Kharel S) came into play in cases of conflicts. All the studies that qualified inclusion criteria were screened for full-text review, which was again done by two reviewers (Kuikel S and Rauniyar R). Overall agreement between the two reviewers was very good (70%–80%).

### 2.2. Data Extraction

Data extraction was done in MS Excel version 2016 by two reviewers (Bista A and Thapaliya S) followed by rechecking of the extracted data by the third reviewer (Kuikel S). Data extraction template was made, and the following data were extracted from each study: author, study year, study design, study population, mean age of study population, the prevalence of stroke in patients with sickle cell anemia, and any interventions done in those patients. We did not have to contact any authors to request the data of their article. The principal data was the prevalence of stroke in patients with sickle cell anemia.

### 2.3. Quality Assessment

Three of the authors analyzed each study using the Joanna Briggs Institute Critical Appraisal tools for use in JBI Systematic Reviews Checklist for Prevalence Studies. It consists of nine yes/no questions that determine if the key components of an observational study are present or not in that study. Two authors (Rauniyar R and Paudel S) assigned scores for each study. A Kappa value was obtained to assess the agreement between the two authors.

### 2.4. Statistical Analysis

In this study, we analyzed the data using STATAV16. Continuous variables are presented as mean and range or as 95% confidence intervals (CI), whereas categorical variables are presented as frequency and proportion (%). We calculated the mean pooled prevalence of stroke in patients with sickle cell anemia, and the outcomes were presented as pooled mean prevalence and 95% CI. A random-effects model was used as this study attempted to generalize findings beyond the included studies to SCA patients all over Asia. Statistical test for publication bias was tested using Egger's regression asymmetry test, and a funnel plot was used to visualize publication bias amongst the studies used for meta-analysis. Sensitivity analysis was carried out by excluding one study at a time (leave-one-out method).

## 3. Results

### 3.1. Study Selection

**Figure 1:**
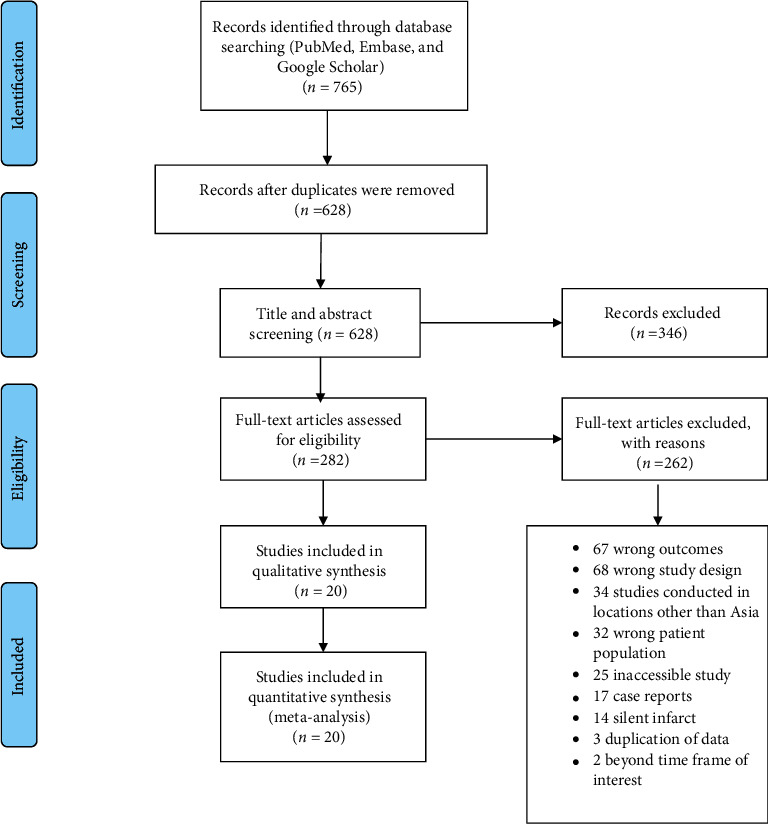
PRISMA flow diagram showing the study retrieval process.

A total of 765 articles were retrieved upon detailed searching in various databases (PubMed, Embase, and Google Scholar). After the removal of 137 duplicate articles, 628 articles were eligible for subsequent literature screening. Upon title and abstract screening, 282 articles were eligible for full-text screening, and 346 articles were excluded. Using the predefined inclusion and exclusion criteria, 20 articles were found to be eligible for including in qualitative and quantitative synthesis. A total of 262 irrelevant articles were excluded after the full-text screening amongst which 67 articles reported outcomes that were not useful in our statistical calculation. The PRISMA flow diagram (Figure 1) depicts the study retrieval process used.

### 3.2. Study Characteristics

The total number of participants from all the included studies (*n* = 20) was 3,956, and the sample size ranged 20–396. Only the studies performed in Asian countries were included, and we were able to obtain eligible studies from Saudi Arabia, India, Pakistan, Turkey, Oman, Kuwait, Yemen, and Lebanon. No studies were available from the remaining Asian countries based on the inclusion criteria. All patients with confirmed sickle cell anemia attending or admitted to various hospitals were the study subjects. The characteristic of individual studies is provided in Table 1.

### 3.3. Prevalence of Stroke

**Figure 2:**
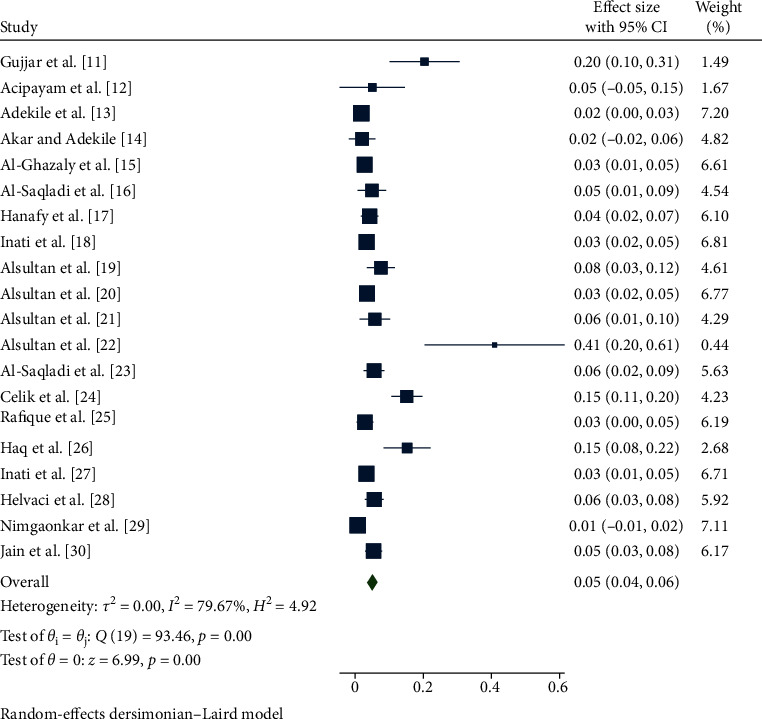
Forest plot showing the pooled prevalence of included studies.

The pooled prevalence of stroke in Asian patients with sickle cell anemia was 5% (95% CI: 4%, 6%) with a range of 1%–41% (Figure 2). Upon subgroup analysis based on country, the prevalence of stroke in sickle cell anemia patients from Turkey, Kuwait, Yemen, Saudi Arabia, Lebanon, Pakistan, and India was found to be 8.90% (95% CI: 2.1%, 15.7%), 1.8% (95% CI: 0.6%, 3%), 4% (95% CI: 2%, 6%), 5% (95% CI: 3.10%, 7%), 3.3% (95% CI: 2%, 4.6%), 8.6% (95% CI: 3.5%, 20.7%), and 2.9% (95% CI: 1.6%, 7.5%), respectively. The pooled prevalence of stroke in sickle cell anemia patients analyzed using articles with <200 and ≥200 sample size was 6.7% (95% CI: 3.5%, 9.9%) and 4.6% (95% CI: 2.8%, 6.4%), respectively. The prevalence obtained from subgroup analysis based on the year of publication was 4.3% (95% CI: 2.6%, 5.9%) for studies published before 2015 and 6.8% (95% CI: 3.1%, 10.4%) for studies published from 2015 to 2020 (Table 2).

### 3.4. Methodological Quality

A Kappa value of 0.634 was obtained that indicates substantial agreement between the two authors for the assessment of the methodological quality of the included study using the JBI scale. The table showing quality assessment is given in Appendix 2.

### 3.5. Sensitivity Analysis

Sensitivity analysis was carried out by excluding one study at a time (leave-one-out method) that showed no significant difference in the prevalence and heterogeneity. The table demonstrating sensitivity analysis is depicted in Appendix 3.

### 3.6. Publication Bias

**Figure 3:**
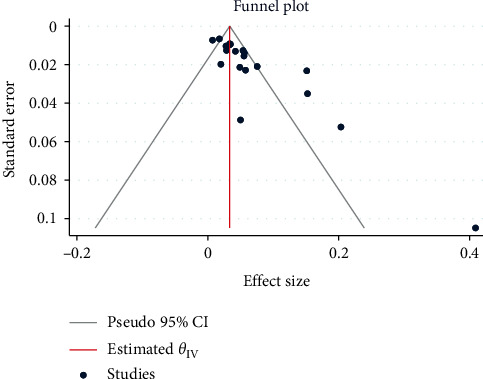
Funnel plot showing the publication bias.

Publication bias was assessed using Egger's regression asymmetry test, and it was statistically insignificant (*p*=0.537) that shows no publication bias.  Figure 3 represents the funnel plot used to visualize publication bias amongst the 20 studies used for meta-analysis.

## 4. Discussion

To our knowledge, this is the first meta-analysis reporting the prevalence of stroke in Asian patients with sickle cell anemia. Our analysis found the prevalence of stroke in sickle cell anemia to be 5% ranging 1%–41% between the studies. This wide range of prevalence reported from studies is due to the interventions (hydroxyurea and blood transfusion either alone or combined) done in the patients with SCA and also due to the variability of the age of the study population.

The prevalence rate reported in our study is in line with a review done in Africa where the prevalence rate of stroke in sickle cell anemia was reported to be 4.2% [31]. Similarly, the results of a study conducted by Powars et al. also resonate with the prevalence rate reported in the results of our study [32]. Moreover, similar prevalence rates of 6.8% have been reported in a study conducted in Uganda [33].

The incidence of stroke is approximately 1.02% per year, and children in the age group of 2–5years are most vulnerable where the possibility of stroke is reported to be very high [34]. Sickle cell anemia leading to stroke can be defined by a potential mechanism of induction of persistent endothelial injury through the effects of profound hypoxia, increased shear stress, irregular endothelial interaction that lead to the formation of sickled red blood cells, and ultimately reperfusion injury-induced inflammation [35].

Sickle cell anemia (SCA) patients are at 221-fold higher risk of experiencing stroke and subsequently 410-fold increased risk of developing cerebral infarction compared with peers of the same age group without the disease. Also, the incidence of ischemic and hemorrhagic strokes is increased in adults with SCD relative to the general population. The prevalence of stroke is 3.75% in patients with SCD. Around 11% of patients have a clinically evident stroke by 20years of age and 24% by 45years of age [36]. Both hemorrhagic and ischemic strokes can occur in patients with sickle cell anemia. Past episodes of transient ischemic attacks, high systolic blood pressure, acute coronary syndrome, and hypoxemia at night time are associated with ischemic strokes [37]. Risk factors for intracranial hemorrhagic stroke in SCA patients include older age, use of corticosteroid or NSAIDs, and immediate blood transfusion (less than 2weeks of the transfusion) [37, 38]. The incidence of stroke is further amplified by several risk factors such as severe malaria, acute or chronic infections, meningitis, and malnutrition, which are highly prevalent in low-income countries [7, 39].

Stroke in children with sickle cell anemia leaves them cognitively impaired compared with their healthy siblings [40]. Secondary prevention is required to escape the devastating consequences of the stroke that are likely to occur in patients with sickle cell anemia. If proper secondary prevention is not taken, up to 70% recurrence rate has been reported for overt ischemic stroke, and the highest risk of recurrence is found to be within 36 months of the initial occurrence of the stroke [41].

Pathologically, elevated cerebral blood velocity confers an increased risk of stroke in sickle cell anemia patients that can be evaluated using transcranial Doppler (TCD) screening [42]. The greater risk of overt stroke in children with sickle cell anemia is recognized by a noninvasive ultrasound procedure, transcranial Doppler [43]. TCD screening is helpful in finding about 90% risk of stroke in asymptomatic children and then successfully managing with maintenance transfusion [43]. The overt stroke risk came down to 1.9% at 18years of age when an early newborn TCD screening and transfusion program was implemented [38]. Transfusion on patients with sickle cell anemia reduces stroke rates along with significant mortality and morbidity. As a result of lesser vaso-occlusive and hemolytic events, the growth and development of the patient is improved [44, 45].

The disease association of stroke with the economic burden is very high in low- and middle-income countries. Effective measures of prevention particularly in vulnerable groups of patients should be implemented along with clinically proven screening techniques to bring down the overall morbidity as well as mortality. Thus, this review highlights the high prevalence of stroke in vulnerable groups of patients that are sickle cell anemia patients, thus necessitating early interventions for both prevention and treatment. This helps reduce both the disease burden on an individual and the economy of the country.

The major strength of this study is that it is the first study exploring the rate of stroke occurrence in sickle cell anemia patients. One of the limitations of our study is not considering the variation in the genotype of hemoglobin in sickle cell anemia that may alter the clinical course and presentation. Other limitations include not taking into consideration the age group of patients with sickle cell anemia as the risk of stroke increases with age [36]. Only a limited number of countries (Oman, Turkey, Kuwait, Yemen, Saudi Arabia, Lebanon, Pakistan, and India) were included in the study due to the unavailability of studies from many countries that met inclusion criteria. We only included studies from Asia, and therefore, findings may not be generalized to populations all over the world.

## 5. Conclusion

Stroke occurrence in sickle cell anemia patients is an emergency complication that needs immediate intervention and management. Though specific interventions are not available to prevent and manage these conditions, treatment options such as hydroxyurea and early and timely blood transfusion based on clinical judgement may help in the reduction of complications like stroke. Many countries in Asia have limited resources and lack adequate tools to identify stroke in patients, especially in pediatrics. Thus, while viewing towards the increasing risk of stroke in sickle cell anemia, clinicians should be aware of this complication.

## Figures and Tables

**Table 1:** Study characteristic table.

Author	Year	Nation	Study design	No. of participants	Mean age (years)	Male	Female
Gujjar et al. [11]	2013	Oman	Cross-sectional	59	13.9 ± 9.5	30	29
Acipayam et al. [12]	2015	Turkey	Cross-sectional	20	21.4 ± 15	8	12
Adekile et al. [13]	2019	Kuwait	Cross-sectional	396	19.2 ± 15.6	206	190
Akar and Adekile [14]	2008	Kuwait	Cross-sectional	50	8.7 ± 2.8	30	20
Al-Ghazaly et al. [15]	2013	Yemen	Cross-sectional	252	12.8 + −9.5	136	105
Al-Saqladi et al. [16]	2007	Yemen	Cross-sectional	102	7.2	56	46
Hanafy et al. [17]	2018	Saudi Arabia	Cross-sectional	237	7.87 ± 3.96	134	103
Inati et al. [18]	2007	Lebanon	Cross-sectional	387	17.9	213	174
Alsultan et al. [19]	2012	Saudi Arabia	Cross-sectional	159	17.8 ± 11.9	77	82
Alsultan et al. [20]	2017	Saudi Arabia	Cross-sectional	376	20	196	180
Alsultan et al. [21]	2014	Saudi Arabia	Cross-sectional	104	38.0 ± 13.3	47	57
Alsultan et al. [22]	2018	Saudi Arabia	Cross-sectional	22	28 + 10.8	8	14
Al-Saqladi et al. [23]	2020	Yemen	Cross-sectional	217	6.9 ± 4.6	103	66
Celik et al. [24]	2015	Turkey	Cross-sectional	238	11.18 ± 4.35	120	118
Rafique et al. [25]	2015	Pakistan	Cross-sectional	175	N/A	85	90
Haq et al. [26]	2019	Pakistan	Cross-sectional	105	5.90 + 3.96	54	51
Inati et al. [27]	2019	Lebanon	Cross-sectional	335	2.9 + −4.5	N/A	N/A
Helvaci et al. [28]	2013	Turkey	Cross-sectional	269	N/A	132	137
Nimgaonkar et al. [29]	2014	India	Cross-sectional	137	14	N/A	N/A
Jain et al. [30]	2010	India	Cross-sectional	316	3.84	194	122

N/A = not available.

**Table 2:** Subgroup analysis.

Subgroups	Total number of studies	Effect size	95% CI	I2 (%)	*p* value
*Sample size*					
<200	10	0.067	0.035–0.099	83.10	0.0001
>200	10	0.046	0.028–0.064	85	0

*Country*					
Saudi Arabia	5	0.05	0.031–0.070	35.98	0
Turkey	3	0.089	0.021–0.157	82.14	0.0098
Yemen	3	0.04	0.020–0.060	29.81	0.0001
Lebanon	2	0.033	0.020–0.046	0	0
Pakistan	2	0.086	0.035–0.207	90.94	0.163
India	2	0.029	0.016–0.075	90.10	0.2057

*Publication date*					
Before 2015	10	0.043	0.026–0.059	70.15	0
2015–2020	10	0.068	0.031–0.104	94.04	0.0003

## Data Availability

All data are available upon reasonable request to the corresponding author.

## Supplementary Materials

Supplementary Materials Search strategy, quality assessment template, sensitivity analysis, and PRISMA checklist of included studies are provided as a supplementary file. Appendix 1: search strategy used in the current systematic review and meta-analysis. Appendix 2: quality assessment of the included articles using the JBI scale. Appendix 3: sensitivity Analysis by omitting one study at a time using the random-effects model. Appendix 4: PRISMA checklist.

## References

[1] Tanabe P., Spratling R., Smith D., Grissom P., Hulihan M. (2019). CE: understanding the complications of sickle cell disease. *American Journal of Nursing*.

[2] Gardner R. V. (2018). Sickle cell disease: advances in treatment. *Ochsner Journal*.

[3] Weatherall D., Akinyanju O., Fucharoen S., Olivieri N., Musgrove P., Jamison D. T., Breman J. G., Measham A. R. (2011). Inherited disorders of hemoglobin. *Disease Control Priorities in Developing Countries*.

[4] Piel F. B., Patil A. P., Howes R. E. (2010). Global distribution of the sickle cell gene and geographical confirmation of the malaria hypothesis. *Natural Communications*.

[5] Adewoyin A. S. (2015). Management of sickle cell anemia: a review for physician education in Nigeria (Sub-Saharan Africa). *Anemia*.

[6] Kirkham F. J., DeBaun M. R. (2004). Stroke in children with sickle cell disease. *Current Treatment Options in Neurology*.

[7] DeBaun M. R., Kirkham F. J. (2016). Central nervous system complications and management in sickle cell disease. *Blood*.

[8] Kwiatkowski J. L., Zimmerman R. A., Pollock A. N. (2009). Silent infarcts in young children with sickle cell disease. *British Journal of Haematology*.

[9] DeBaun M. R., Armstrong F. D., McKinstry R. C., Ware R. E., Vichinsky E., Kirkham F. J. (2012). Silent cerebral infarcts: a review on a prevalent and progressive cause of neurologic injury in sickle cell anemia. *Blood*.

[10] Hashmi A. A., Al Hashmi A., Aaron S. (2020). Acute ischemic stroke in sickle cell anemia challenges for thrombolysis. *Dubai Medical Journal*.

[11] Gujjar A. R., Zacharia M., Al-Kindi S. (2013). Transcranial Doppler ultrasonography in sickle cell disease. *Journal of Pediatric Hematology/oncology*.

[12] Acipayam C., Oktay G., Ilhan G., Çürük M. A. (2015). Hemoglobin SE disease in Hatay, in the southern part of Turkey. *Thalassemia Reports*.

[13] Adekile A. D., Al-Sherida S., Marouf R., Mustafa N., Thomas D. (2019). The sub-phenotypes of sickle cell disease in Kuwait. *Hemoglobin*.

[14] Akar N. A., Adekile A. (2008). Ten-year review of hospital admissions among children with sickle cell disease in Kuwait. *Medical Principles and Practice*.

[15] Al-Ghazaly J., Al-Dubai W., Abdullah M., Al-Mahagri A., Al-Gharasi L. (2013). Characteristics of sickle cell anemia in Yemen. *Hemoglobin*.

[16] Al-Saqladi A.-W., Delpisheh A., Bin-Gadeem H., Brabin B. J. (2007). Clinical profile of sickle cell disease in Yemeni children. *Annals of Tropical Paediatrics*.

[17] Hanafy E., Al Atawi Y., Al Balawi A., Al Atawi G., Salama M., Ahmed N. (2018). Prevalence of stroke in patients with sickle cell anemia, a single center’s experience. *EC Neurology*.

[18] Inati A., Jradi O., Tarabay H. (2007). Sickle cell disease: the Lebanese experience. *International Journal of Laboratory Hematology*.

[19] Alsultan A., Aleem A., Ghabbour H. (2012). Sickle cell disease subphenotypes in patients from Southwestern Province of Saudi Arabia. *Journal of Pediatric Hematology/oncology*.

[20] Alsultan A., Jastaniah W., Al Afghani S. (2016). Demands and challenges for patients with sickle-cell disease requiring hematopoietic stem cell transplantation in Saudi Arabia. *Pediatric Transplantation*.

[21] Alsultan A., Alabdulaali M. K., Griffin P. J. (2014). Sickle cell disease in Saudi Arabia: the phenotype in adults with the Arab-Indian haplotype is not benign. *British Journal of Haematology*.

[22] Alsultan A., Al-Suliman A. M., Aleem A., AlGahtani F. H., Alfadhel M. (2018). Utilizing whole-exome sequencing to characterize the phenotypic variability of sickle cell disease. *Genetic Testing and Molecular Biomarkers*.

[23] Al-Saqladi A. W., Maddi D. M., Al-Sadeeq A. H. (2020). Blood transfusion frequency and indications in Yemeni children with sickle cell disease. *Anemia*.

[24] Celik T., Unal S., Ekinci O. (2015). Mean platelet volume can predict cerebrovascular events in patients with sickle cell anemia. *Pakistan Journal of Medical Sciences*.

[25] Rafique M., Zia S., Khan M. A., Alqahtani Y. A. M., Huneif M. A. (2015). Demographic and clinical characteristics of children with sickle cell disease. *Pakistan Pediatric Journal*.

[26] Haq S., Ahmad T., Liaqat I., Ali S., Haq Z. (2019). Frequency of childhood ischemic stroke in children presenting with sickle cell anemia. *Med Forum*.

[27] Inati A., Al Alam C., El Ojaimi C. (2019). Sickle cell disease burden in North Lebanon. *Blood*.

[28] Helvaci M. R., Ayyildiz O., Gundogdu M. (2013). Gender differences in severity of sickle cell diseases in non-smokers. *Pakistan Journal of Medical Sciences*.

[29] Nimgaonkar V., Krishnamurti L., Prabhakar H., Menon N. (2014). Comprehensive integrated care for patients with sickle cell disease in a remote aboriginal tribal population in southern India. *Pediatric Blood & Cancer*.

[30] Jain D., Italia K., Sarathi V., Ghoshand K., Colah R. (2012). Sickle cell anemia from central India: a retrospective analysis. *Indian Pediatrics*.

[31] Noubiap J. J., Mengnjo M. K., Nicastro N., Kamtchum-Tatuene J. (2017). Neurologic complications of sickle cell disease in Africa. *Neurology*.

[32] Powars D., Wilson B., Imbus C., Pegelow C., Allen J. (1978). The natural history of stroke in sickle cell disease. *The American Journal of Medicine*.

[33] Munube D., Katabira E., Ndeezi G. (2016). Prevalence of stroke in children admitted with sickle cell anaemia to Mulago Hospital. *BMC Neurology*.

[34] Ohene-Frempong K., Weiner S. J., Sleeper L. A. (1998). Cerebrovascular accidents in Sickle cell anemia: rates and risk factors. *Blood*.

[35] Hoppe C. (2005). Defining stroke risk in children with sickle cell anaemia. *British Journal of Haematology*.

[36] Earley C. J., Kittner S. J., Feeser B. R. (1998). Stroke in children and sickle-cell disease: baltimore-Washington cooperative young stroke study. *Neurology*.

[37] Strouse J. J., Lanzkron S., Urrutia V. (2011). The epidemiology, evaluation and treatment of stroke in adults with sickle cell disease. *Expert Review of Hematology*.

[38] Switzer J. A., Hess D. C., Nichols F. T., Adams R. J. (2006). Pathophysiology and treatment of stroke in sickle-cell disease: present and future. *The Lancet Neurology*.

[39] Connes P., Verlhac S., Bernaudin F. (2013). Advances in understanding the pathogenesis of cerebrovascular vasculopathy in sickle cell anaemia. *British Journal of Haematology*.

[40] Ashley-Koch A., Murphy C. C., Khoury M. J., Boyle C. A. (2001). Contribution of sickle cell disease to the occurrence of developmental disabilities: a population-based study. *Genetics in Medicine*.

[41] Stotesbury H., Kawadler J. M., Hales P. W., Saunders D. E., Clark C. A., Kirkham F. J. (2019). Vascular instability and neurological morbidity in sickle cell anemia: an integrative framework. *Frontiers in Neurology*.

[42] Adams R. J., McKie V. C., Carl E. M. (1997). Long-term stroke risk in children with sickle cell disease screened with transcranial Doppler. *Annals of Neurology*.

[43] Adams R. J., McKie V. C., Hsu L. (1998). Prevention of a first stroke by transfusions in children with sickle cell anemia and abnormal results on transcranial Doppler ultrasonography. *New England Journal of Medicine*.

[44] Wang W. C., Morales K. H., Scher C. D. (2005). Effect of long-term transfusion on growth in children with sickle cell anemia: results of the STOP trial. *The Journal of Pediatrics*.

[45] Lezcano N. E., Odo N., Kutlar A., Brambilla D., Adams R. J. (2006). Regular transfusion lowers plasma free hemoglobin in children with sickle-cell disease at risk for stroke. *Stroke*.

