# Supplementary material for: Prevalence of Stroke in Asian Patients with Sickle Cell Anemia: A Systematic Review and Meta-Analysis
Source: Neurol Res Int. 2021 Jun 3;2021:9961610. doi: 10.1155/2021/9961610 (PMC8195673; doi:10.1155/2021/9961610)

**Supplementary Content**

**Prevalence of Stroke in Asian patients with sickle cell anemia: A systematic review and meta analysis**

Appendix 1: Search strategy used in the current systematic review and meta- analysis.

Appendix 2: Quality assessment of the included articles using JBI scale

Appendix 3: Sensitivity Analysis by omitting one study at a time using random effect model

Appendix 4: PRISMA checklist

This supplementary material has been provided by the authors to give readers additional information**.**

**Appendix 1:**

Embase search Strategy:

Date of Search: December 5, 2020

Session Results

.......................................................

No. Query Results Results Date

#12. #9 AND #10 AND [english]/lim AND [humans]/lim AND 438 5 Dec 2020

[2000-2020]/py

#11. #9 AND #10 501 5 Dec 2020

#10. 'china' OR 'india' OR 'indonesia' OR 'pakistan' 8,359,074 5 Dec 2020

OR 'bangladesh' OR 'japan' OR 'philippines' OR

'iran' OR 'thailand' OR 'myanmar' OR 'iraq' OR

'afghanistan' OR 'saudi arabia' OR 'uzbekistan'

OR 'malaysia' OR 'yemen' OR 'nepal' OR 'north

korea' OR 'sri lanka' OR 'kazakhstan' OR 'syrian

arab republic' OR 'cambodia' OR 'jordan' OR

'azerbaijan' OR 'united arab emirates' OR

'tajikistan' OR 'israel' OR 'laos' OR 'lebanon'

OR 'kyrgyzstan' OR 'turkmenistan' OR 'singapore'

OR 'oman' OR 'kuwait' OR 'georgia republic' OR

'mongolia' OR 'armenia' OR 'qatar' OR 'bahrain'

OR 'timor-leste' OR 'cyprus' OR 'bhutan' OR

'maldives' OR 'brunei darussalam' OR 'viet nam'

OR 'turkey republic' OR 'south korea' OR 'taiwan'

OR 'hong kong' OR 'asia'

#9. #7 AND #8 3,813 5 Dec 2020

#8. #1 OR #2 OR #5 640,337 5 Dec 2020

#7. #3 OR #4 OR #6 43,131 5 Dec 2020

#6. 'hemoglobin s'/exp 5,624 5 Dec 2020

#5. stroke 484,126 5 Dec 2020

#4. 'sickle cell anemia' 38,355 5 Dec 2020

#3. 'sickle cell anemia'/exp 40,265 5 Dec 2020

#2. 'brain ischemia'/exp 192,650 5 Dec 2020

#1. 'cerebrovascular accident'/exp 336,288 5 Dec 2020

Appendix 2: Quality assessment of included studies using JBI scale

| Author | 1. Was the sample frame appropriate to address the target population? | 2. Were study participants sampled in an appropriate way? | 3. Was the sample size adequate? | 4. Were the study subjects and the setting described in detail? | 5. Was the data analysis conducted with sufficient coverage of the identified sample? | 6. Were valid methods used for the identification of the condition? | 7. Was the condition measured in a standard, reliable way for all participants? | 8. Was there appropriate statistical analysis? | 9. Was the response rate adequate, and if not, was the low response rate managed appropriately? | Total Score |
| --- | --- | --- | --- | --- | --- | --- | --- | --- | --- | --- |
| Gujjar et al | Y | Y | N | Y | Y | U | Y | Y | Y | 7 |
| Acipayam et al | Y | Y | Y | Y | Y | U | Y | Y | Y | 8 |
| Adekile et al. | Y | Y | Y | Y | Y | U | Y | Y | Y | 8 |
| Akar et al. | Y | Y | Y | Y | Y | U | Y | Y | Y | 8 |
| Al-Ghazaly et al. | Y | Y | Y | Y | Y | U | Y | Y | Y | 8 |
| Al-Saqladi et al. | Y | Y | Y | Y | Y | U | Y | Y | Y | 8 |
| Hanafy et al. | Y | Y | Y | Y | Y | U | Y | Y | Y | 8 |
| Inati et al. | N | Y | Y | Y | Y | U | Y | Y | Y | 7 |
| Alsultan et al. | N | Y | Y | Y | Y | U | Y | Y | Y | 7 |
| Alsultan et al. | Y | Y | Y | Y | Y | U | Y | Y | Y | 8 |
| Alsultan et al. | Y | Y | Y | Y | Y | U | Y | Y | Y | 8 |
| Alsultan et al. | Y | Y | Y | Y | Y | U | Y | Y | Y | 8 |
| Al-Saqladi et al. | Y | Y | Y | Y | Y | U | U | Y | Y | 7 |
| Celik et al. | Y | Y | Y | Y | Y | U | Y | Y | Y | 8 |
| Rafique et al. | Y | Y | Y | Y | Y | U | Y | Y | Y | 8 |
| UlHaq et al. | Y | Y | Y | Y | Y | U | Y | Y | Y | 8 |
| Inati et al. | Y | Y | Y | Y | Y | U | Y | Y | Y | 8 |
| Helvaci et al. | Y | Y | Y | Y | Y | U | Y | Y | Y | 8 |
| Nimgaonkar et al. | Y | Y | Y | Y | Y | U | Y | Y | Y | 8 |
| Jain et al. | Y | Y | Y | Y | Y | U | Y | Y | Y | 8 |

Y= yes, N= No, U= Unclear

Appendix 3: Sensitivity Analysis of Included studies

| Omitting studies | Pooled prevalence | LCI | UCI | I2 |
| --- | --- | --- | --- | --- |
| None | 0.05 | 0.036 | 0.064 | 79.67 |
| Gujjar et al. 2013 | 0.047 | 0.033 | 0.06 | 78.29 |
| Acipayam et al. 2015 | 0.05 | 0.036 | 0.064 | 80.27 |
| Adikile et al. 2019 | 0.053 | 0.038 | 0.068 | 79.17 |
| Najwa Ali Akar | 0.052 | 0.037 | 0.066 | 80.64 |
| Al-Ghazaly 2013 | 0.054 | 0.038 | 0.07 | 81.64 |
| Al-Saqladi 2007 | 0.05 | 0.036 | 0.064 | 80.63 |
| Hanafy 2018 | 0.051 | 0.036 | 0.065 | 80.64 |
| Inati 2007 | 0.052 | 0.037 | 0.067 | 80.74 |
| Alsultan 2012 | 0.048 | 0.034 | 0.063 | 79.86 |
| Alsultan 2017 | 0.052 | 0.037 | 0.067 | 80.74 |
| Alsultan 2014 | 0.05 | 0.035 | 0.064 | 80.5 |
| Alsultan 2018 | 0.047 | 0.034 | 0.061 | 77.67 |
| Al-Saqladi 2020 | 0.05 | 0.035 | 0.064 | 80.31 |
| Celik 2015 | 0.043 | 0.031 | 0.056 | 73.29 |
| Rafique 2015 | 0.052 | 0.037 | 0.066 | 80.71 |
| UlHaq 2019 | 0.046 | 0.033 | 0.06 | 78.02 |
| Inati 2019 | 0.052 | 0.037 | 0.067 | 80.74 |
| Helvaci 2013 | 0.05 | 0.035 | 0.064 | 80.18 |
| Nimgaonkar 2014 | 0.053 | 0.039 | 0.067 | 76.95 |
| Jain 2010 | 0.05 | 0.035 | 0.064 | 80.17 |

Appendix 4: PRISMA Checklist


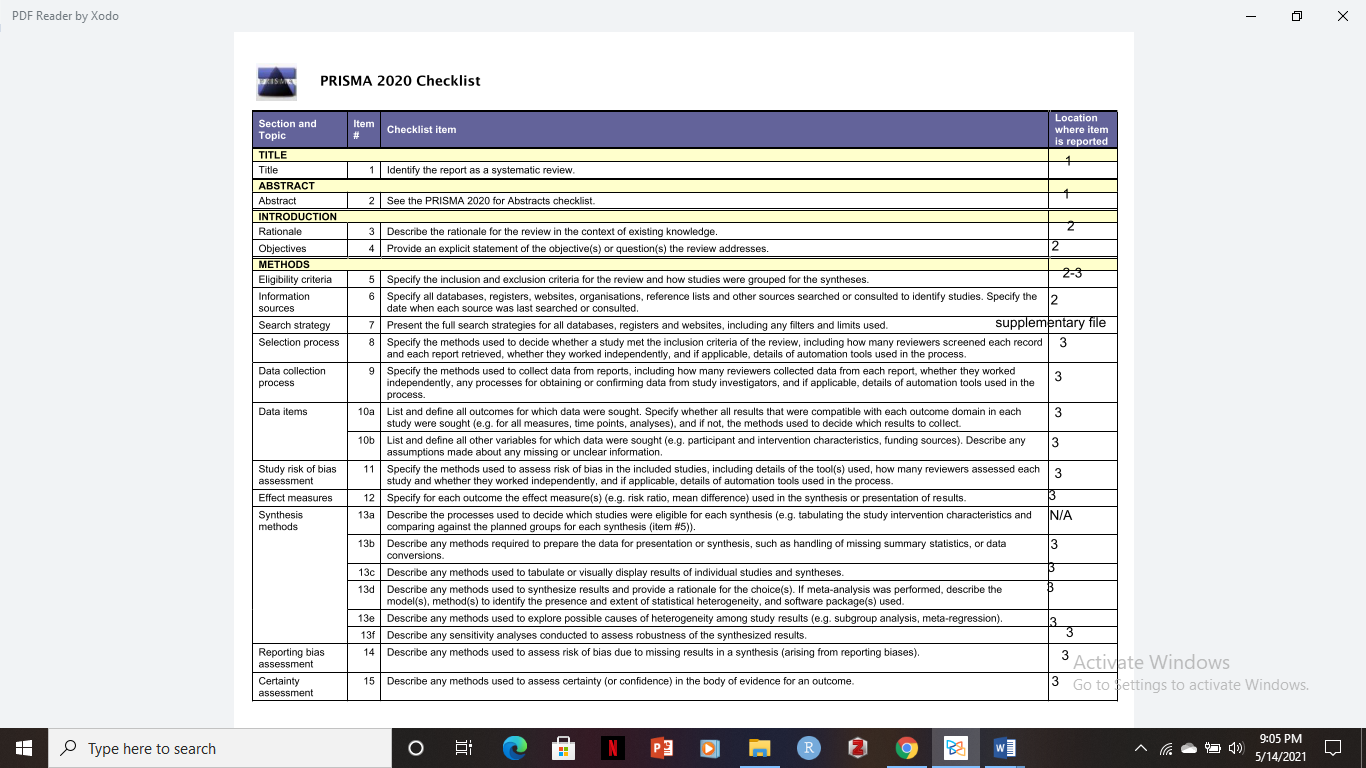


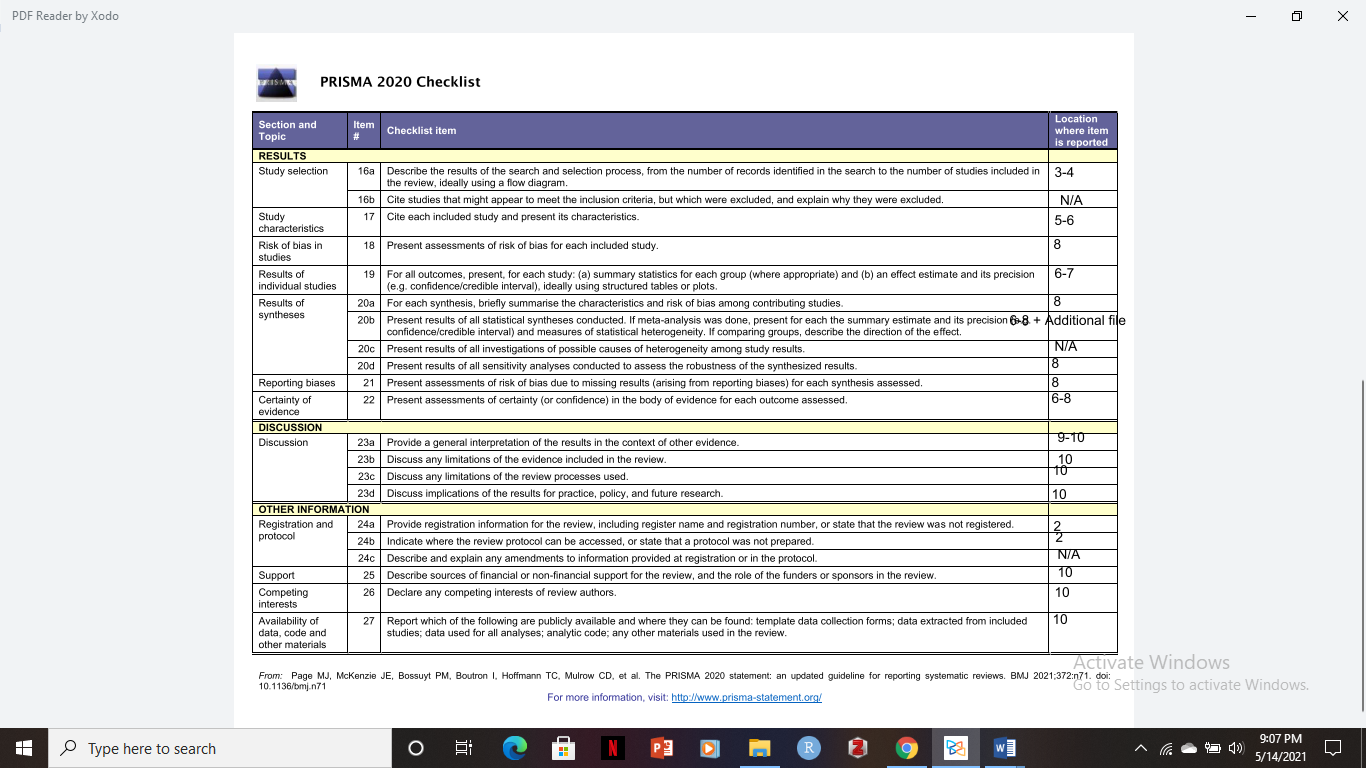

Supplement: Supplementary Materials — Search strategy, quality assessment template, sensitivity analysis, and PRISMA checklist of included studies are provided as a supplementary file. Appendix 1: search strategy used in the current systematic review and meta-analysis. Appendix 2: quality assessment of the included articles using the JBI scale. Appendix 3: sensitivity Analysis by omitting one study at a time using the random-effects model. Appendix 4: PRISMA checklist. [file 9961610.f1.docx]
